# Advances in green nanotechnology: Data for green synthesis and characterization of iron nanoparticles synthesized using *Galinsoga parviflora, Conyza bonariensis* and *Bidens pilosa* leaf extracts, and their application in degradation of methylene blue dye and rifampicin antibiotic

**DOI:** 10.1016/j.dib.2022.108882

**Published:** 2023-01-11

**Authors:** Sammy Indire Wanakai, Patrick Gachoki Kareru, David Sujee Makhanu, Edwin Shigwenya Madivoli

**Affiliations:** a Chemistry Department, Jomo Kenyatta University of Agriculture and Technology, P.O. Box 62, 000-00200, Nairobi, Kenya; b Department of Biological & Physical Sciences, Karatina University, P.O. Box 1957-10101, Karatina, Kenya

**Keywords:** Data, Green synthesis, Iron Nanoparticles, Organic pollutants, Degradation, BpNPs, *Bidens pilosa* iron nanoparticles, CbNPs, *Conyza bonariensis* iron nanoparticles, GpNPs, *Galinsoga parviflora* iron nanoparticles

## Abstract

This data article reports contents of the information derived from an efficient, environmentally friendly, and low-cost method of synthesis and recovery of iron nanoparticles using *Galinsoga parviflora, Conyza bonariensis* and *Bidens pilosa* aqueous leaf extracts as reducing, stabilizing, and capping agents, and applications of the nanoparticles in degradation of organic dyes and antibiotics. Various spectroscopic and microscopic techniques were used to collect the data. Data is displayed in the form of .raw files, graphs, images, Microsoft Excel sheets, .data point files, and PDF files, along with other formats. Data analysis and interpretation methods have also been presented. Researchers, research students, academicians, and industrialists can benefit greatly from the data in order to gain knowledge about the green synthesis of iron nanoparticles and related applications such as degradation organic pollutants. The data is deposited in the mendeley data repository as two independent datasets at https://data.mendeley.com/datasets/rxkv6j7hrx.

**Specifications Table**

**Table:** 

Subject	NanoChemistry, Environmental Science, Environmental Chemistry.
Specific subject area	Green Chemistry, Nanotechnology, Nano remediation.
Type of data	Excel files, data files, images, text files, figures, tables, graphs, PDF files
How data was acquired	Ultraviolet Visible Spectrum (UV/VIS) (UV 1800, Shimadzu), Fourier Transform Infrared Spectrophotometer (FTS 8000, Japan), X-ray Fluorescence (Bruker, X-ray Powder Diffractometer (XRD) (STOE STADIP and Cie GmbH, Darmstadt, Germany), and Scanning Electron Microscope (SEM) (FEI XL30 Sirion FEG, Oxford Instruments Plc, Abingdon, United Kingdom),
Data format	Raw, analyzed, and filtered
Description of data collection	Samples were collected, left to dry, milled and extracted with distilled water to obtain the aqueous extracts. The extracts were then used in the synthesis with 0.1 M iron III chloride solution. The iron nanoparticles were dried prior to further characterization. 1. UV Vis data for Synthesis of GpNPs Iron oxide nanoparticles. Xlsx – Data for Synthesis pf GpNPs Iron oxide nanoparticles 2. UV Vis data-CBNPs synthesis.xlsx – Data for synthesis of CbNPs Iron oxide nanoparticles. 3. UV Vis for Green synthesis of Bidens pilosa nps.xlsx – Data for synthesis of BpNPs Iron oxide nanoparticles. 4. UV Vis Scan of cb, bp, gp extract-PXKMUU.xlsx. – UV Visible spectra of the extracts. 5. UV VIS data and UV Vis spectra ferric chloride extracts and nanoparticles – Data for the spectra of ferric chloride extracts and nanoparticles. 6. TOTAL FLAVONOIDS.pdf – Data for flavonoids. 7. Supplementary Materials.docx – Data for spectra for UV Visible and FTIR summary. 8. STANDARD –Total Flavonoids.pdf – Data for the standards Total Flavonoids. 9. sem images of CbNPs, GpNPs, BpNPs.zip – Data for the SEM Images of the iron nanoparticles. 10. QUANTIFICATION OF TOTA PHENOLS.pdf – Data for the Total phenols. 11. FTIR DATA.rar – Fourier Transform Spectrophotometer data for the iron nanoparticles 12. Deg by cbnps.xlsx – Data for degradation of methylene blue using CbNPs 13. Copy of Updated FTIR DATA Iron CB, BP, GP NANOPARTICLES.xlsx – Fourier Transform Spectrophotometer data for the iron nanoparticles. 14. cbnpsh2o2raf.deg.xlsx – UV Vis data for degradation of rifampicin using CbNPs 15. cbnpsbasicalone.xlsx. – UV Vis data for degradation of rifampicin using CbNPs at basic media. 16. cbnpsrafh2o2aloinedeg.xlsx – UV Vis data for degradation of rifampicin using CbNPs and hydrogen peroxide alone. 17. Basic deg. Alone.xlsx – UV Vis data for degradation of rifampicin using CbNPs in the presence of the base alone. 18. baseand h2o2.xlsx – spectra of base and hydrogen peroxide solution. 19. Cce. Dosage, leo, pH temps, temps – data files for the spectra of degradation of the organic compounds at different concentration, dosage, pH, and temperatures.
Data Source location	JKUAT, Nairobi (Kenya), Fribourg (Switzerland)
Data accessibility	All data have been deposited in the Mendeley Data public repository, and can be accessed via the following link: Direct URL to data: https://data.mendeley.com/datasets/rxkv6j7hrx

## Value of the Data

• The data can be used to comprehend the UV/VIS absorption spectra, including storage and temperature stability, as well as the XRD and FTIR spectra of the synthesized iron nanoparticles.
• There has been no previous research on the synthesis of iron nanoparticles using aqueous extracts of *Galinsoga parviflora, Conyza bonariensis*, and *Bidens pilosa*.
• The findings present a new low-cost method for recovering synthesized iron nanoparticles and the possibility of using iron nanoparticles as catalysts in the degradation of methylene blue dye and rifampicin antibiotic and the kinetics involved in the mechanism of degradation.
• The data can be extremely beneficial to academicians, research students, researchers, and industrialists seeking to learn about the green synthesis of iron nanoparticles and their catalytic nature in degradation of dyes and pharmaceutical compounds (organic compounds).
• The data obtained by replicating the protocols presented can be used to develop other experiments in the field of green synthesis and catalytic degradation of organic pollutants.

## 1 Objective

The objective of this dataset/paper is to provide a simple and safe way that can be employed to synthesize iron nanoparticles using leaf extracts of Galinsoga parviflora, Conyza bonariensis and Bidens pilosa as well as any other plant. Also, the data can help in providing steps for the synthesis and characterization of the synthesized iron nanoparticles using various techniques, such as X-ray diffraction (XRD), Fourier-transform infrared (FT-IR) spectroscopy, transmission electron microscopy (TEM) and scanning electron microscopy (SEM). Through this data one can also use the steps as a guide to study the effect of the leaf extracts on the size and shape of the iron nanoparticles. Finally, the data can offer an insight in studying of the efficacy of the synthesized iron nanoparticles in degrading the methylene blue dye and rifampicin antibiotic, investigation of the mechanism of degradation of methylene blue dye and rifampicin antibiotic by the synthesized iron nanoparticles, and explore the potential applications of the synthesized iron nanoparticles in environmental remediation.

## 2 Data Description

This information came from the synthesis and characterization of highly stable iron nanoparticles derived from aqueous extracts of *Galinsoga parviflora, Conyza bonariensis*, and *Bidens pilosa* leaves. Because of the previously reported unique activities of plant extracts, the preparation of iron nanoparticles from these plants, particularly with water, may offer unique advantages over those from other solvents [1]. The information presented here is made up of images, Microsoft Excel data sheets, and tables, among other items. The following information is provided:

### 2.1 Data for Quantitative Phytochemical Analysis (Total Phenolic and Flavonoid Contents) of *Galinsoga parviflora, Conyza bonariensis*, and Bidens *pilosa* Leaves

(i) Raw data of the Quantitative Analysis of *Galinsoga parviflora, Conyza bonariensis*, and *Bidens pilosa* leaves.
(ii) Tables of the quantitative total phenolic and total flavonoid content of *Galinsoga parviflora, Conyza bonariensis*, and *Bidens pilosa* leaves.

### 2.2 Data for Characterization of Iron Nanoparticles from Aqueous Extracts of *Galinsoga parviflora, Conyza bonariensis*, and Bidens *pilosa* Leaves

(i) Images of the green synthesis protocol of iron nanoparticles.
(ii) Raw data and images of the UV – visible absorption spectra of iron III chloride, *Galinsoga parviflora, Conyza bonariensis*, and *Bidens pilosa* leaf extracts, and their nanoparticles from 200 to 250 nm range.
(iii) Raw data and images of the FTIR spectra of *Galinsoga parviflora, Conyza bonariensis*, and *Bidens pilosa* leaf extracts, and their nanoparticles from 4000 to 400 cm^−1^.
(iv) Raw data for the EDXRF, XRD and SEM as well as the analysis report.

### 2.3 Data for Degradation of Methylene Blue

(i) Raw data/data points of the degradation of methylene blue using *Galinsoga parviflora, Conyza bonariensis*, and *Bidens pilosa* iron nanoparticles/hydrogen peroxide, and hydrogen peroxide alone.
(ii) Images of the plots of $\ln\frac{A}{A_{0}}$ (where A is the final absorbance and A_0_ the final absorbance) for the kinetics of methylene blue degradation using *Galinsoga parviflora, Conyza bonariensis*, and *Bidens pilosa* iron nanoparticles/hydrogen peroxide, and hydrogen peroxide alone.

### 2.4 Data for Degradation of Rifampicin Antibiotic

(i) Raw data/data points of the degradation of rifampicin antibiotic using *Galinsoga parviflora, Conyza bonariensis*, and *Bidens pilosa* iron nanoparticles/hydrogen peroxide, and hydrogen peroxide alone.
(ii) Images of the plots of $\ln\frac{A}{A_{0}}$ (where A is the final absorbance and A_0_ the final absorbance) for the kinetics of rifampicin antibiotic degradation using *Galinsoga parviflora, Conyza bonariensis*, and *Bidens pilosa* iron nanoparticles/hydrogen peroxide, and hydrogen peroxide alone.

## 3 Experimental Design, Materials and Methods

### 3.1 Collection of Plant Samples

Fresh leafs of *Galinsoga Parviflora, Bidens pilosa* and *Conyza bonariensis* were collected from Bungoma East Sub- County, Bungoma County, Kenya in January 2018. the leafs were transported to JKUAT Botanical laboratory for identification.

### 3.2 Plant Material Preparation

The plants were then washed thoroughly with running tap water and rinsed with distilled water, cut into small parts, and air-dried at room temperature (20–25 °C) for 14 days to remove moisture. After drying, the plant materials were ground finely using a milling machine [2].

### 3.3 Sample Preparation for Green Synthesis of Iron Nanoparticles

20 g of the dried and ground sample of *Galinsoga parviflora, Conyza bonariensis* and *Bidens pilosa* were weighed into different labeled 250 mL volumetric flasks and 200 mL distilled water added and the mixture boiled at 70 °C with constant shaking for 45 min. The extracts were cooled and filtered three times with cotton wool and further with Whatman No.1 filter paper to get a clear extract [3].

### 3.4 Preparation of 0.1 M FeCl_3_ Solution

The working solution 0.1 M FeCl_3_ .6H_2_O was prepared by weighing 13.52 g FeCl_3_ .6H_2_O and dissolving in 100 mL of distilled water and topping to 500 mL with enough distilled water [4].

### 3.5 Synthesis of Iron Nanoparticles Using Leaf Extracts

Synthesis was performed by adding 30 mL of plant extracts to 10 mL of 0.1 M Ferric Chloride dropwise using a burette with constant stirring using a magnetic stirrer at room temperature and pressure [5]. A black precipitate was formed which was washed several times with distilled water, centrifuged at 6000 rpm for 10 min using a centrifuge (model, Centurion 6000) and the obtained nanoparticles were dried at 60 °C in the oven [6].

### 3.6 Characterization of the Iron Nanoparticles

The plant extracts were characterized using UV –Vis, and FT – IR, while the green synthesized nanoparticles were characterized by UV –Vis, FT – IR, XRF, XRD, and SEM techniques to determine the absorption band of the chromophores in the extract, the functional groups involved in the reduction and stabilization of the nanoparticles, the elemental composition of the nanoparticles, the crystallinity and morphology of the iron nanoparticles.

### 3.7 UV – Vis Analysis of the Extracts and Formation of Iron Nanoparticles

The aqueous extracts of *Galinsoga parviflora, Conyza bonariensis* and *Bidens pilosa* and the synthesized nanoparticles were scanned in the range of 200 – 800 nm using a UV – Vis spectrophotometer (UV 1800, Shimadzu) in order to confirm the formation of the nanoparticles and distilled water was used as a blank.

### 3.8 Functional Group Analysis of the Extract and Iron Nanoparticles

FT – IR analysis was performed on the extracts and the nanoparticles using Shimadzu FT – IR spectrophotometer (FTS—8000, Japan) to confirm the functional groups involved in the reduction and stabilization of the nanoparticles. The method employed involved use of potassium bromide (KBr) method where the samples were prepared by grinding the extract or nanoparticles with KBr in the ratio of 1:10. 10 mg of the samples and 100 mg of KBr (FT –IR grade) were ground in a mortar and pestle and pellets prepared by pressing under a pressure of 75 kNcm^−2^ for 3 min, the spectral resolution set at 4 cm^–1^ and the scanning range from 400 to 4000 cm^–1^ [7]. The FT – IR spectra manifested from the *Galinsoga parviflora, Conyza bonariensis* and *Bidens pilosa* extracts and the green synthesized nanoparticles were recorded and the minor and major peaks identified.

### 3.9 X-ray Fluorescence Analysis

The elemental composition of the iron nanoparticles was evaluated using X-ray fluorescence machine Bruker model

### 3.10 Crystalline Size Determination Using XRD

The average crystalline size of the iron nanoparticles was determined using X – Ray powder Diffraction (STOE & Cie GmbH, Darmstadt, Germany), the X-ray generator equipped with a copper tube operating at 40 kV and 40 mA and the sample irradiated with a monochromatic Cu Kα radiation with a wavelength of 1.5409 nm and 2θ range of 2–90 °C at 0.05 °C intervals.

### 3.11 Scanning Electron Microscope Micrographs

To report the surface morphology of the nanoparticles, morphological analysis was performed using Tescan Mira3 LM FE Scanning electron microscope, Germany operated at an accelerating voltage of 3 Kv, and to avoid charging effect, the samples were gold sputtered before observation.

## 4 Catalytic Activity of the Synthesized Iron Nanoparticles

### 4.1 Reagent Preparation

1 mL of methylene blue was diluted to 50 ml with distilled water.

### 4.2 Catalytic Degradation of Methylene Blue

The ability of the iron nanoparticles to catalyze degradation of methylene blue dye was investigated. Hydrogen peroxide and the nanoparticles were employed for this study. Catalytic activity of hydrogen peroxide and the nanoparticles was evaluated as controls by scanning then in the range of 200 – 800 nm using double-beam UV–Vis spectrophotometer (UV 1800, Shimadzu).

Two spectral scans were done on 3 mL of methylene solution in the range of 200–800 nm in 5 min intervals using double-beam UV–Vis spectrophotometer (UV 1800, Shimadzu). Thereafter, 50 mg of iron nanoparticles (*Galinsoga parviflora, Conyza bonariensis* and *Bidens pilosa* iron nanoparticles separately) were sprinkled on the solution and allowed to scan for $10\frac{1}{2}\;$h in 5 min interval. The various scans were performed using double-beam UV–Vis spectrophotometer (UV 1800, Shimadzu) and recorded in excel sheet. Percentage degradation was obtained and the kinetic study of degradation was calculated using a plot of $\ln\frac{A}{A_{0}}$ against time.

### 4.3 FT – IR Spectra of Nanoparticles After Degradation

FTIR spectra of *Conyza bonariensis* iron nanoparticles (CbNPs), *Galinsoga parviflora* iron nanoparticles (GpNPs) and *Bidens pilosa* iron nanoparticles (BpNPs) after degradation of methylene blue dye was performed to determine any shifts and changes in the intensity of peaks.

### 4.4 Catalytic Degradation of Rifampicin

The ability of the iron nanoparticles to degrade rifampicin was determined using methods employed before [8].

(i) **Preparation of 10** **mg/L (12.15** **µM) Rifampicin Solution**

0.0025 g of rifampicin was dissolved in 2 mL of methanol and 10 mL of distilled water added to 250 mL volumetric flask and the contents topped to the mark by washing the contents in the beaker with distilled water.

### 4.5 Degradation Studies

The experiment was performed using 10 mg iron oxide nanoparticles, 12.15 µM rifampicin, and 250 µL of 0.25 µM H_2_O_2_ solution. 3 mL of rifampicin was scanned in the range of 600 to 350 nm in intervals of 2 min using a UV –Vis spectrophotometer, 1800, Shimadzu Corporation, Kyoto, Japan. Further, 250 µL of 0.25 µM hydrogen peroxide was added and two spectral scans, then 50 mg of the iron nanoparticles were added into the solution and more scans were taken [9].

### 4.6 Degradation of Rifampicin Varying the Adsorbent Dose, Temperatures, Time and pH of the Reaction Media

(i) **Variation of pH**

Degradation studies were performed at pH 3, 7, and 12, where the pH of the reaction media was adjusted using 0.1 M NaOH and 0.1 M HCl.

(ii) **Variation of Temperature**

The effect of the reaction temperature on the rate of degradation of rifampicin was evaluated at 25, 40, 50, and 60 °C. In brief, 3 mL of the rifampicin sample was mixed with 1 mg of the nanoparticle and boiled at the various temperatures (25, 40, 50, and 60 °C). Thereafter, the thermodynamic parameters associated with degradation of rifampicin were determined based on the linear form of the Van't Hoff equation:

$$\ln K_{eq}=\;\frac{\Delta H}{RT}+\frac{\Delta S}{R}$$

where ∆H and ∆S denote the enthalpy, and entropy of the degradation reaction, while R is the universal gas constant, and T refers to the temperature (kelvin) [10,11].

(iii) **Variation of Adsorbent Dose**

The influence of the amount of iron nanoparticles on the rate of degradation of rifampicin was studied using 20, 10, 5, 1 mg of CbNPs, GpNPs, and BpNPs samples.

(iv) **Variation of Time**

Time taken for rifampicin to degrade when using Varying the Adsorbent Dose, Temperatures, and pH of the Reaction Media was performed by recording the initial and the final time taken for complete degradation.

(v) **Degradation Efficiency**

The removal efficiency of the nanoparticles against rifampicin was calculated using the equation below where *C_0_* is the initial concentration of rifampicin and *C_t_* is the final rifampicin concentration at time *t* in minutes [12]*.*

$$Degradation\;efficiency\;\left(\%\;\right)=\;\frac{C_{0}-C_{t}}{C_{0}}\;x\;100$$

(vi) **Reaction Order**

The order of the reaction for the degradation of rifampicin was determined experimentally by plotting the data assuming a first–order reaction using the equation;

$$ln{\left[A\right]}_{t}=\;-kt+\ln{\left[A\right]}_{0}$$

and a second-order reaction using the equation;

$$\frac{1}{{\left[A\right]}_{t}}=\;-kt+\frac{1}{{\left[A\right]}_{0}}$$

From these plots, the reaction order was determined from the best line of fit obtained from the graphs and the results tabulated after calculation [10,^11^,13].

### 4.7 Thermodynamic Parameters

The thermodynamic parameters associated with rifampicin degradation which include entropy change and enthalpy change of the degradation system were determined based on the linear form of the Van't Hoff equation:

$$\ln K_{eq}=\;\frac{\Delta H}{RT}+\frac{\Delta S}{R}$$

where ∆S and ∆H are the entropy and enthalpy of the degradation reaction, while R is the universal gas constant, and T is the temperature (kelvin) [10,11].

### 4.8 FT – IR Analysis of the Iron Nanoparticle After Degradation of Rifampicin

The infrared spectra of iron oxide nanoparticles before and after degradation of rifampicin under different reaction conditions was determined using the KBr method stated before.

## Ethics Statement

No human subjects were employed through this study.

## CRediT authorship contribution statement

**Sammy Indire Wanakai:** Conceptualization, Methodology, Software, Data curation, Writing – original draft, Writing – review & editing, Visualization, Investigation, Software, Validation. **Patrick Gachoki Kareru:** Visualization, Investigation, Supervision, Writing – review & editing. **David Sujee Makhanu:** Conceptualization, Methodology, Data curation, Writing – original draft, Visualization, Investigation, Supervision, Validation, Writing – review & editing. **Edwin Shigwenya Madivoli:** Conceptualization, Methodology, Data curation, Writing – original draft, Visualization, Investigation, Software, Validation, Writing – review & editing.

## Declaration of Competing Interest

The authors declare that they have no competing interests.

## Data Availability

Data for Green Synthesis of Iron Nanoparticles Using Galinsoga parviflora, Conyza bonariensis and Bidens pilosa leaf extracts, and their Application in Degradation of Organic pollutants (Original data) (Mendeley Data)
